# Supplementary material for: Learning curve and surgical outcome of robotic assisted colorectal surgery with ERAS program
Source: Sci Rep. 2022 Nov 29;12:20566. doi: 10.1038/s41598-022-24665-w (PMC9709162; doi:10.1038/s41598-022-24665-w)
Supplement: Supplementary file 1 — Supplementary Information 1. [file 41598_2022_24665_MOESM1_ESM.zip › LSC Raw data-Ñ[▒K/094/P_ñw╝╨░O▒Kñσ.pdf]

[redacted]

Pathologic diagnosis:
1. Gallbladder, laparoscopic cholecystectomy --- Chronic cholecystitis with cholelithiasis and cholesterolosis.
2. Ascending to transverse colon, terminal ileum, and appendix, right hemicolectomy --- a. Adenocarcinoma, moderately differentiated, of transverse colon. Tumor invaded to serosa and metastasized to mesocolic lymph nodes (1/27). The appendix, ileocecal valve, and bilateral cut ends are free of tumor. b. Status post T-loop colostomy with stitch granuloma and xanthgranulomatous inflammation of stoma site, mesocolon, and omentum. c. Fibrous adhesion to small intestine.

已電子簽章

Ancillary study for diagnosis:
IHC stains for PMS2 and MSH6 are done on section G. Both markers are preserved.

敬告：本單僅供醫師參考使用，不具任何申請效力！！
請病患妥為保管，若不慎遺失需自負個人資料外洩責任！

Prognostic and predictive factor:
1. Tumor size: 3.5x3.2 cm.
2. Depth of invasion: Visceral peritoneum (including tumor continuous with serosal surface through area of inflammation).
3. Histologic grade: G2: Moderately differentiated.
4. Angiolymphatic invasion: Present.
5. Perineural invasion: Present.
6. Tumor deposits (discontinuous extramural extension): Not identified.
7. Macroscopic tumor perforation: Not identified.
8. Surgical Margins:
a) Proximal Margin: Uninvolved by invasive carcinoma, distance of tumor from margin: 35 cm.
b) Distal Margin: Uninvolved by invasive carcinoma, distance of tumor from margin: 2.7 cm.
c) Radial or Mesenteric Margin: Uninvolved by invasive carcinoma, distance of tumor from margin: 1.5 cm.
9. Lymph node status: Involved (1/27).
10. Extranodal involvement: Not identified.
11. Tumor regression grading S/P CCRT: No known presurgical therapy.
12. Type of polyp in which invasive carcinoma arose: Not identified.
13. Additional pathologic findings: Not identified.
14. Pathological TNM Stage: pT4aN1a (According to the eighth edition, American Joint Committee on Cancer Staging Guidelines for Tumors).
15. TNM descriptors: Not applicable.

Gross description:
The specimen consists of 1) a piece of opened gallbladder, 4.8x1.7x1.1 cm, with wall thickness, 0.3 cm. No stone or polyp is seen inside. 2) a segment of colon, 25 cm in length, and 5.2 cm in distal cut end circumference, and a segment of terminal ileum, 10 cm in length, and 2.5 cm in cut end circumference. An unremarkable appendix, 4.3 cm, is noted. A gray tan firm annular elevated and centrally ulcerative tumor, 3.5x3.2 cm, is noted, 2.7 cm to the distal cut end. On cross section, the tumor invaded to the mesocolon with 5 mm to the radial margin grossly, and serosal retraction is also noted. Several tan and soft tumor nodules, up to 3x2.6x1 cm, is seen over colon serosa and omentum, with suspected fibrous adhesion to radial resection margin. A segment of intestine, 6 cm in length and 2 cm in diameter, is seen attached to the cecal serosa by tan and soft tumor nodules. The appendix, ileocecal valve, and both cut ends are free of tumor. Representative part for section: A) specimen 1 B-T) specimen 2 [B: ileal cut end, C: colon cut end, D: appendix, E-J: main tumor, K-M: transverse colon tumor nodule and radial margin, N-P: omental nodule, Q: ileocecal valve, R: cecum and small intestine adhesion site, S-T: lymph nodes].

Note:
1. The interpretation of MMR panel and recommended studies are as below: No loss of nuclear expression of MMR proteins: low probability of microsatellite instability-high (MSI-H). Please note: There are exceptions to the above IHC interpretations. These results should not be considered in isolation, and clinical correlation with genetic counseling is recommended to assess the need for germline testing.
2. This case has been peer reviewed by two doctors.
#T-59420_2 #M-81403_2 2 1105 S0000F
#T-C4560_2 #M-81406_2 2 1105 000000
#T-59200_2 #M-09183_2 0 1105 000000
#T-59308_2 #M-18800_2 0 1100 000000
#T-63000_2 #M-30010_2 0 1100 000000
#T-63000_2 #M-55250_2 0 1100 000000
